# Rice Yellow Mottle Virus stress responsive genes from susceptible and tolerant rice genotypes

**DOI:** 10.1186/1471-2229-8-26

**Published:** 2008-03-03

**Authors:** Marjolaine Ventelon-Debout, Christine Tranchant-Dubreuil, Thi-Thu-Huang Nguyen, Martine Bangratz, Christelle Siré, Michel Delseny, Christophe Brugidou

**Affiliations:** 1 UMR5096, IRD 911 Avenue Agropolis, BP54501, 34394 Montpellier, France; 2 UMR5096, Université de Perpignan 52, Avenue de Villeneuve, 66860 Perpignan Cedex, France

## Abstract

**Background:**

The effects of viral infection involve concomitant plant gene variations and cellular changes. A simple system is required to assess the complexity of host responses to viral infection. The genome of the Rice yellow mottle virus (RYMV) is a single-stranded RNA with a simple organisation. It is the most well-known monocotyledon virus model. Several studies on its biology, structure and phylogeography have provided a suitable background for further genetic studies. 12 rice chromosome sequences are now available and provide strong support for genomic studies, particularly physical mapping and gene identification.

**Results:**

The present data, obtained through the cDNA-AFLP technique, demonstrate differential responses to RYMV of two different rice cultivars, i.e. susceptible IR64 (*Oryza sativa indica*), and partially resistant Azucena (*O. s. japonica*). This RNA profiling provides a new original dataset that will enable us to gain greater insight into the RYMV/rice interaction and the specificity of the host response. Using the SIM4 subroutine, we took the intron/exon structure of the gene into account and mapped 281 RYMV stress responsive (RSR) transcripts on 12 rice chromosomes corresponding to 234 RSR genes. We also mapped previously identified deregulated proteins and genes involved in partial resistance and thus constructed the first global physical map of the RYMV/rice interaction. RSR transcripts on rice chromosomes 4 and 10 were found to be not randomly distributed. Seven genes were identified in the susceptible and partially resistant cultivars, and transcripts were colocalized for these seven genes in both cultivars. During virus infection, many concomitant plant gene expression changes may be associated with host changes caused by the infection process, general stress or defence responses. We noted that some genes (e.g. ABC transporters) were regulated throughout the kinetics of infection and differentiated susceptible and partially resistant hosts.

**Conclusion:**

We enhanced the first RYMV/rice interaction map by combining information from the present study and previous studies on proteins and ESTs regulated during RYMV infection, thus providing a more comprehensive view on genes related to plant responses. This combined map provides a new tool for exploring molecular mechanisms underlying the RYMV/rice interaction.

## Background

Numerous analyses of the virus-induced transcriptome in different host plants have been reported. These studies generally catalogued a set of induced changes to virus infection (reviewed in [1]). The most consistent observation is that viruses, in compatible host-virus interactions, trigger a range of stress responses, including defence response genes. Perturbations of different signalling pathways in the host are induced by change in gene expression involved in some specific interactions between virus and host proteins, as well as gene expression that are not directly involved in these interactions.

The *Rice yellow mottle virus *(RYMV, Sobemovirus) is the most damaging pathogen of rice, endemic to the Africa where it is widespread. It has a simple genomic organisation with a single-stranded RNA encoding four open reading frames (ORF). Its biology and structure are well known [2,3]. The diversification as well as the phylogeography of this virus are well documented [4,5]. Different levels of resistance against the RYMV have been described in the cultivated Asian and African rice species, *O. sativa *and *O. glaberrima *[6], and a partial resistance has been observed in some upland *japonica *rice cultivars [7]. A major recessive resistance gene located on chromosome 4, coding for an eIF(iso)4G protein, has been recently characterized [8]. Genetic basis of RYMV partial resistance has been studied using a doubled haploid population (IR64 (*O. s. indica*, highly susceptible to RYMV) × Azucena (*O. s. japonica*, partially resistant to RYMV) and showed seven quantitative trait loci involved in this partial resistance [7,9]. This virus is thus a very attractive model since genomic studies on this pathogen give a well-known background, and open up horizon for other rice viruses.

The rice genome has been both well mapped genetically and physically [10,11]. Genomes of the varieties Nipponbare (*O. sativa japonica*) and 93-11 (*O. s. indica*) have been integrally sequenced [12-20], and these 12 rice chromosomes are available online [21]. A large number of predicted genes correspond to putative proteins with unknown function and a portion of the genome corresponds to repetitive sequences [22,23]. Nevertheless, it appears interesting to predict putative function of a gene based on homology searches, thus enabling the identification of candidate genes involved in a particular biochemical pathway, like virus resistance genes involved in the mechanisms developed by plant to resist virus infection.

We have already published a global protein profiling in rice cell suspension infected with RYMV, using 2-D gel electrophoresis. We also identified proteins (defence and stress related proteins, translation and protein turnover, and metabolic proteins) with altered accumulation in response to the virus in susceptible and partially resistant rice cultivars [24]. Moreover our first study on transcriptome of both susceptible and partially resistant rice cultivars infected with RYMV identified variations of gene expression in defence, metabolic and photosynthesis pathways [25]. These results, compared to others studies, indicate that similar events are happening at the protein and the mRNA levels. In this article, we proceed to a fine study of the transcriptome and report the location of 281 RYMV stress responsive sequences (RSR sequences identified by cDNA-AFLP). These RSR genes belong to different functional classes notably defence, photosynthesis pathway, and metabolism and have been mapped onto rice chromosomes using SIM4 subroutine that aligns cDNA fragment against genomic sequence. We present here the first compiled physical map for virus/Rice interaction with the model RYMV/Rice.

## Results

### Using cDNA-AFLP to discover genes induced or repressed during virus infection

**Figure 1:**
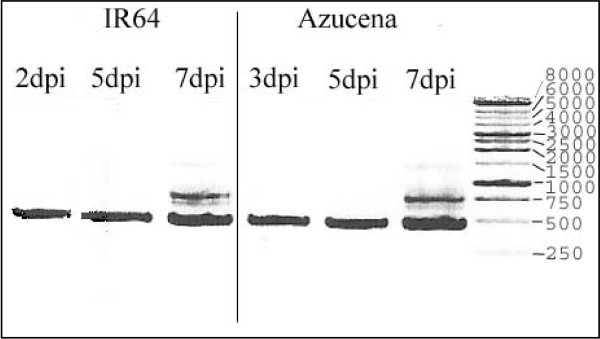
**Quantification of RYMV RNA by RT-PCR in IR64 and Azucena inoculated leaves**. The size of the amplified fragments has been predicted at 439 bp. The lanes on the agarose gel were loaded with: reaction products from RNAs of IR64 infected leaves harvested at 2, 5, and 7 dpi, RNAs of Azucena infected leaves harvested at 3, 5, and 7 dpi, 1 kb ladder (Promega).

**Figure 2:**
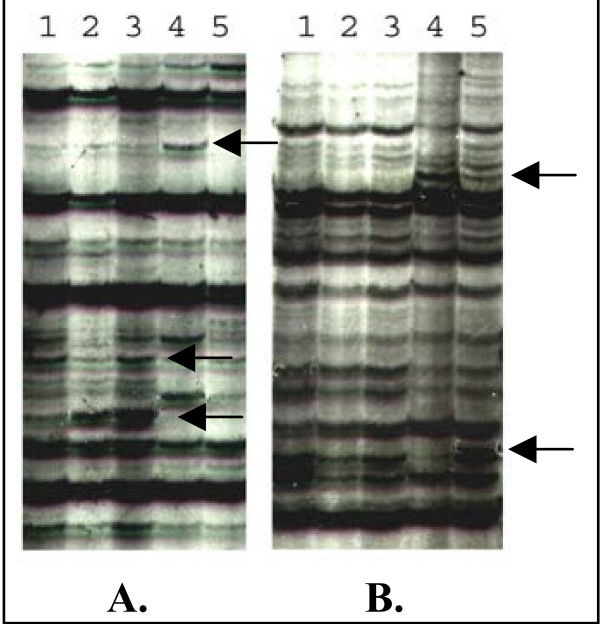
**Azucena (A.) and IR64 (B.) cDNA-AFLP display from non-stressed leaves (lane 1), wounded leaves (lane 2), and RYMV inoculated leaves harvested at 3 dpi for Azucena/2 dpi for IR64 (lane 3), 5 dpi (lane 4) and 7 dpi (lane 5).** This five lanes set represented amplification by one particular primer combination. Arrows represented differential bands.

**Figure 3:**
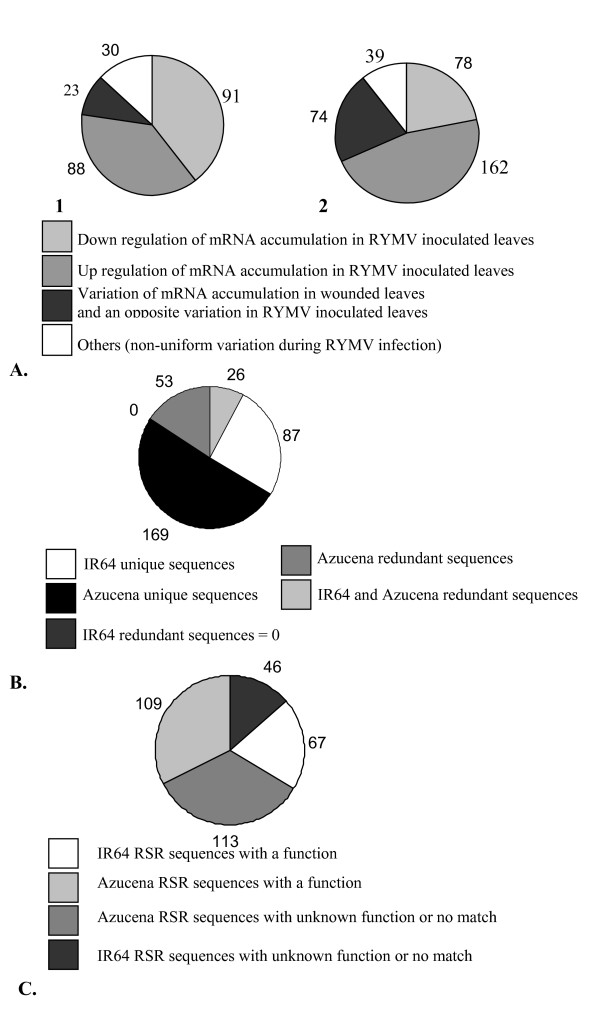
**A**. **Overall results of cDNA-AFLP analysis, representing the variation of mRNA accumulation in both IR64 (1) and Azucena (2) rice cultivars under RYMV infection.B**. RSR sequences redundancy. **C**. RSR sequences annotation.

We analyzed the transcript profiling using the cDNA-AFLP technique on two different rice cultivars: IR64 (*O. s. indica*) highly susceptible to the RYMV, and Azucena (*O. s. japonica*) tolerant to the RYMV. We looked at different time points after infection: 2, 5, 7 days post inoculation (dpi) for IR64 and 3, 5, 7 dpi for Azucena. The kinetics gave a similar pattern of virus content in infected leaves of both cultivars (Figure 1). Non infected IR64 and Azucena leaves were used as control at the first point of the kinetic for the both cultivars, and IR64 and Azucena wounded leaves without RYMV were harvested at the first point of the kinetic for internal control since the virus infection was mechanically done. A section of a typical AFLP gel is shown on Figure 2. Changing patterns of gene expression were revealed using more than 20 000 cDNA fragments from both IR64 and Azucena cultivars. The resulting AFLP products ranged in length from 40 base pairs (bp) to 500 bp, and 60 to 100 bands were observed for each of the 256 primer combinations on the gel. Only bands with a present-absent pattern were considered. The cDNA-AFLP screen identified 353 cDNA fragments corresponding to differentially accumulated transcripts during the first week of RYMV infection for Azucena, and 232 cDNA fragments for IR64. One hundred sixty two (46%) of the AFLP-cDNA fragments corresponded to up-regulated transcripts and 78 (22%) to down-regulated transcripts for Azucena, and 74 (21%) to wound-regulated and RYMV-regulated transcripts (transcripts with a variation of accumulation observed in wounded leaves and an opposite variation observed in infected leaves). On the contrary, 88 (38%) of cDNA fragments corresponded to up-regulated transcripts for IR64, 91 (39%) to down-regulated transcripts, and only 23 (10%) to wound-regulated and RYMV-regulated transcripts (Figure 3).

### Compilation of sequences from induced and altered AFLP fragments

The recovery from the acrymalide gel and the reamplification were tricky steps and thus we were not able to get a clean sequence for all cDNA fragments. We obtained 113 (49% out of the 232 fragments) and 222 clean sequences (63% out of the 353 fragments) for IR64 and Azucena respectively. These sequences were designated RYMV Stress Responsive (RSR) transcripts. After cutting off adaptators and vector sequences, all the sequences showed a length between 20 and 300 bp. All the 335 RSR sequences (113 for IR64 and 222 for Azucena) have been referenced in GenBank (the GenBank accession numbers are from DQ883824 to DQ884159) and have been blasted against each other. Eighty seven IR64 and 169 Azucena RSR sequences match only once in GenBank Database and were named IR64 or Azucena unique sequences, respectively. Fifty three Azucena sequences matched more than once and were named Azucena redundant sequences. Twenty six IR64 and Azucena sequences matched among each other and are named IR64 and Azucena redundant sequences (in this case one IR64 sequences matched to one or more Azucena sequences, and vice versa) (Figure 3B). These 79 RSR redundant sequences actually corresponded to 34 unique genes. This redundancy is directly due to the technique used: since cDNA-AFLP is based on amplification of fragments of cDNA, we expected to identify several fragments of a single cDNA.

All the 335 RSR sequences were blasted against the NR database (All non-redundant GenBank CDS translations + RefSeq Proteins + PDB + SwissProt + PIR + PRF). Sixty-seven (59%) IR64 and 109 (49%) Azucena sequences matched with an accession. We used a low strengency, an E-value < 10^-4^, as lengths of the sequences were small. Forty-six (41%) IR64 and 113 (51%) Azucena sequences did not match or were similar to sequences with unknown function (Table 1, Figure 3C). A large part of the AFLP sequences did not have an allocated function, and this might be due to the short size of the AFLP sequences and the quality of the NR database. Among these unclassified sequences, 29 were redundant within 12 clusters of sequences.

**Table 1:** RSR sequences were blasted against the public database Swissprot using blast subroutine [39] and mapped onto rice chromosomes.

	**IR64**	**Azucena**
**Total number of RSR**	232	353
RSR sequences (% compared to total number of RSR)	113 (49%)	222 (63%)
Non-redundant RSR sequences (% compared to RSR sequences)	105 (93%)	196 (88%)
RSR sequences with no match (% compared to RSR sequences)	46 (41%)	113 (51%)
RSR sequences with a match E-value < 10^-4 ^(% compared to RSR sequences)	67 (59%)	109 (49%)

**Mapped onto rice chromosomes**		
RSR sequences mapped onto rice chromosomes	101	183

All the RSR sequences were classified into functional categories (see additional file 1). We observed some variation of mRNA accumulation in each functional class for the both cultivars. Our results revealed important changes in transcript underlying that viral infection generated numerous changes in both susceptible and partially resistant cultivars. The major deregulated functional classes were defence, signalling, metabolism and photosynthesis. We also noticed 5 different RSR sequences encoding transposons/retroelements negatively regulated during the progress of infection in Azucena (DQ884156, DQ883989, DQ883835, DQ883911, and DQ883916).

### RSR sequences mapped onto rice chromosomes

All cDNA-AFLP fragments were mapped and aligned against the release 5 of the twelve chromosomes of *Oryza sativa *[21,26]. The map is available on the IRD platform website [see Availabilities section]. Two hundred eighty four RSR sequences (183 from Azucena and 101 from IR64) were physically mapped. Fifty-one RSR sequences have not been mapped since the score was too low to map them with enough confidence. Multiple RSR sequences mapped at more than one location and multiple RSR sequences mapped at the same localisation and thus corresponded to the same gene. We mapped respectively 46, 40, 54, 38, 25, 30, 35, 26, 20, 32, 26, and 26 RSR sequences onto the 12 rice chromosomes. We identified 20 RSR sequences from the both cultivars mapped at the same location and 27 and 22 positions were characterized with more than one Azucena RSR sequences and IR64 RSR sequences, respectively (Table 2). These redundant sequences corresponded to the same gene and were not used to perform the statistical test to study the distribution of RSR genes along the chromosomes. In order to study whether the RSR gene distribution along each chromosome corresponded to the distribution of genes, we compared the distribution of RSR sequences and the distribution of ATG codon corresponding to the gene distribution. Then we used the contingency test performed for each chromosome with an alpha error of 0.5%. The distribution of RSR sequences on the chromosomes 4 and 10 were significantly different from the ATG codon distribution.

**Table 2:** Azucena and IR64 RSR sequences mapped at the same location. a: cDNA-AFLP fragment GenBank accession.

**ID^a^**	**Cultivar**	**Variation of gene expression^b^**	**Description^c^**	**Chromosome**	**Start position (bp)**
gb|DQ883945	Azucena	(7dpi)+	sphingosine-1-phosphate lyase	1	37379
gb|DQ883961	Azucena	(2dpi)+	sphingosine-1-phosphate lyase	1	37386
gb|DQ884107	IR64	(BI)-(2,5,7dpi)-	sphingosine-1-phosphate lyase	1	37386
gb|DQ884097	IR64	(BI)-(2dpi)+	ATP-binding cassette	1	661520
gb|DQ883934	Azucena	(5,7dpi)-	ABC transporter	1	661520
gb|DQ883962	Azucena	(BI)+(3dpi)+	ABC transporter	1	7762693
gb|DQ884118	IR64	(2dpi)+	ABC transporter	1	7762700
gb|DQ883963	Azucena	(5,7dpi)+	ABC transporter	1	7762703
gb|DQ884067	IR64	(5,7dpi)-	OsTATC	1	25926035
gb|DQ883887	Azucena	(5,7dpi)-	OsTATC	1	25926035
gb|DQ883980	Azucena	(BI)+	transcription factor EREBP1	2	2730720
gb|DQ883981	Azucena	(BI)+	transcription factor EREBP1	2	2730722
gb|DQ883986	Azucena	(3dpi)+	transcription factor EREBP1	2	2730722
gb|DQ884016	Azucena	(5,7dpi)-	potassium transporter HAK1p	2	5509064
gb|DQ884017	Azucena	(7dpi)+	potassium transporter HAK1p	2	5509070
gb|DQ883933	Azucena	(5,7dpi)-	peroxisomal targeting signal 2 receptor	2	8205239
gb|DQ883935	Azucena	(5dpi)+	peroxisomal targeting signal 2 receptor	2	8205240
gb|DQ883937	Azucena	(5dpi)+	-	2	22063567
gb|DQ884129	IR64	(5dpi)+	-	2	22063567
gb|DQ884022	IR64	(BI)-(2,5)-	senescence-associated protein	2	28710373
gb|DQ884021	IR64	(5,7dpi)-	senescence-associated protein	2	28710376
gb|DQ884026	IR64	(7dpi)+	-	3	8112599
gb|DQ884027	IR64	(5dpi)+	-	3	8112604
gb|DQ883936	Azucena	(3dpi)+	-	3	19981332
gb|DQ884126	IR64	(5,7dpi)+	-	3	19981332
gb|DQ883998	Azucena	(BI)+(3,5,7dpi)+	splicing factor	3	28989563
gb|DQ883999	Azucena	(BI)-(3,5,7dpi)-	splicing factor	3	28989563
gb|DQ883950	Azucena	(3,5,7dpi)+	inositol phosphate kinase	3	29491859
gb|DQ883952	Azucena	(BI)+	inositol phosphate kinase	3	29491859
gb|DQ884093	IR64	(5,7dpi)-	inositol phosphate kinase	3	29492176
gb|DQ884005	Azucena	(BI)+(3,5,7dpi)+	receptor kinase	3	34628274
gb|DQ884006	Azucena	(5dpi)+	receptor kinase	3	34628277
gb|DQ884124	IR64	(2,5,7dpi)+	receptor kinase	3	34628284
gb|DQ884032	IR64	(5,7dpi)-	-	4	4985354
gb|DQ884031	IR64	(5,7dpi)-	-	4	4985360
gb|DQ883901	Azucena	(3,5,7dpi)-	large ribosomal protein 23	4	9131675
gb|DQ883900	Azucena	(BI)-(3dpi)-	large ribosomal protein 23	4	9131683
gb|DQ883941	Azucena	(5dpi)+	-	4	25607867
gb|DQ883946	Azucena	(5dpi)+	-	4	25607880
gb|DQ884135	IR64	(BI)+(2,5dpi)+	photosystem II stability/assembly factor	6	156408
gb|DQ884136	IR64	(BI)+(2dpi)+	photosystem II stability/assembly factor	6	156408
gb|DQ883985	Azucena	(BI)-	-	6	294284
gb|DQ883988	Azucena	(3,5,7dpi)-	-	6	294284
gb|DQ884103	IR64	(BI)+(2,5,7dpi)+	NADH dehydrogenase subunit 4	6	3064929
gb|DQ884104	IR64	(BI)+(2,5,7dpi)+	NADH dehydrogenase subunit 4	6	3064929
gb|DQ883896	Azucena	(7dpi)+	-	7	8998816
gb|DQ883909	Azucena	(5,7dpi)+	-	7	8998816
gb|DQ884103	IR64	(BI)+(2,5,7dpi)+	NADH dehydrogenase subunit 4	7	12659627
gb|DQ884104	IR64	(BI)+(2,5,7dpi)+	NADH dehydrogenase subunit 4	7	12659627
gb|DQ883901	Azucena	(3,5,7dpi)-	large ribosomal protein 23	7	14240632
gb|DQ883900	Azucena	(BI)-(3dpi)-	large ribosomal protein 23	7	14240640
gb|DQ884133	IR64	(5,7dpi)+	-	8	501007
gb|DQ884134	IR64	(7dpi)+	-	8	501007
gb|DQ883901	Azucena	(3,5,7dpi)-	large ribosomal protein 23	8	9254046
gb|DQ883900	Azucena	(BI)-(3dpi)-	large ribosomal protein 23	8	9254054
gb|DQ884076	IR64	(5,7dpi)+	OSH45	8	11757217
gb|DQ884077	IR64	(5dpi)-	OSH45	8	11757217
gb|DQ884022	IR64	(BI)-(2,5dpi)-	senescence-associated protein	9	7658
gb|DQ884021	IR64	(5,7dpi)-	senescence-associated protein	9	7661
gb|DQ883901	Azucena	(3,5,7dpi)-	large ribosomal protein 23	9	14504325
gb|DQ883900	Azucena	(BI)-(3dpi)-	large ribosomal protein 23	9	14504333
gb|DQ884103	IR64	(BI)+(2,5,7dpi)+	NADH dehydrogenase subunit 4	10	10583266
gb|DQ884104	IR64	(BI)+(2,5,7dpi)+	NADH dehydrogenase subunit 4	10	10583266
gb|DQ883842	Azucena	(5,7dpi)+	-	11	1188582
gb|DQ884055	Azucena	(5,7dpi)+	-	11	1188589
gb|DQ884103	IR64	(BI)+(2,5,7dpi)+	NADH dehydrogenase subunit 4	12	21873723
gb|DQ884104	IR64	(BI)+(2,5,7dpi)+	NADH dehydrogenase subunit 4	12	21873723
gb|DQ883842	Azucena	(5,7dpi)+	-	12	26262316
gb|DQ884055	Azucena	(5,7dpi)+	-	12	26262323

b: pattern of deregulation, inside brackets BI for buffer inoculated control or *n *dpi (*n *days post inoculation), + for up regulation, - for down regulation. c: putative function as found after blasting against NR database.

### Validation of variation of gene expression by quantitative RT-PCR

**Figure 4:**
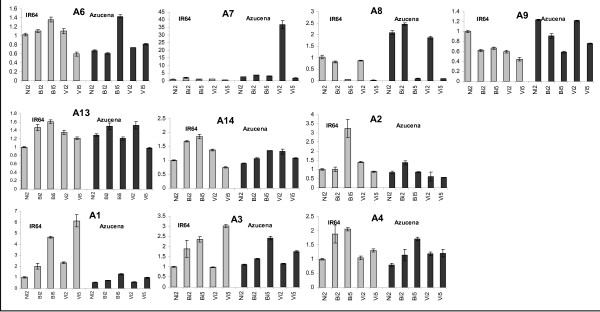
**Quantitative RT-PCR results.** Mean of the 3 replicates, and standard error. (NI: non inoculated; BI2: buffer inoculation, harvest at 2dpi; BI5: buffer inoculation, harvest at 5dpi; VI2: virus inoculation, harvest at 2dpi; VI5: virus inoculation, harvest at 5dpi – grey diagram: IR64; black diagram: Azucena). **A6**: IR64-gb|DQ884097/Azucena-gb|DQ883871. **A7**: Azucena-gb|DQ884155. **A8**: IR64-gb|DQ884049. **A9**: IR64-gb|DQ884066. **A13**: Azucena-gb|DQ883861. **A14**: IR64-gb|DQ884107/Azucena-gb|DQ883961. **A2**: IR64-gb|DQ884118/Azucena-gb|DQ883962. RSR sequences from IR64 and Azucena which co localized: **A1**: IR64-gb|DQ884103/Azucena-gb|DQ883886. **A3**: IR64-gb|DQ884067/Azucena-gb|DQ883887. **A4**: IR64-gb|DQ884124/Azucena-gb|DQ884005.

cDNA-AFLP results were confirmed by quantitative RT-PCR using a subset of 10 genes as probes (Figure 4). All the corresponding genes showed a variation of expression using RT-PCR and thus confirming that they were not some false positives. We did not observe exactly the same pattern of variation of gene expression; nevertheless the quantitative RT-PCR always confirmed that genes identified by cDNA-AFLP were actually deregulated during RYMV infection. The quantitative RT-PCR also gave a more precise picture of the time course of changing. Thus, we identified a short-lived up-expression at 2 dpi of the gene gb|DQ884155 which encodes for a putative S-receptor kinase. Moreover, the quantitative RT-PCR analysis revealed that the susceptible cultivar seemed to have a higher degree of response to the injury than the tolerant cultivar: variations of gene expression (up- or down-regulation) were more important for the wounded leaves of the susceptible cultivar.

## Discussion

Viruses use a variety of strategies to promote their infection in plant. Large studies have already mentioned the various impacts of virus infection [27]. Well-documented modifications of host cells have already been reported [[1] for review]. Suppression of post-transcriptional gene silencing is one of the strategies to promote their infection [28]. Under viral infection, there are many concomitant plant gene variations and cellular changes [29]. The effects of virus infection remain complex and causal relationships are still difficult to establish. Moreover, major traits of host responses and the specificity of a susceptible compared to a tolerant response remain still unclear.

As the response to virus infection involves biological quantitative traits and has multi-factorial inheritance, a global analysis of the multiple genes that are affected during the RYMV infection appears to be a straightforward approach for the study of interactions between virus and host. Using two dimensional electrophoresis and ESTs analysis, we have already established a complex pattern of protein and gene regulations, and identified numerous changes in cell balance involved in host response ([25,24]; data not shown). We identified at both transcriptional and post transcriptional levels some variations in the photosynthesis pathway and metabolism as potential clues of the susceptible response. Here we analyzed the transcript profiling using the cDNA-AFLP technique on the same two rice cultivars: IR64 (*O. s. indica*) highly susceptible to the RYMV, and Azucena (*O. s. japonica*) tolerant to the RYMV. This technique has been shown to be a powerful gel-based genome-scaled transcript profiling [30] and allowed us to identify a large number of genes involved in rice-RYMV interaction during the first week of infection.

We observed more regulations of host gene expression for the tolerant cultivar than for the susceptible one (Figure 3A). We noticed an up-regulation of expression of a gene encoding a photosystem II stability assembly factor (RSR sequences DQ884135 and DQ884136) for the cultivar IR64 during the kinetic of RYMV infection, and a down regulation of expression of gene encoding a photosystem II phosphoprotein for the cultivar Azucena under RYMV infection (DQ884058). These results were consistent with our previous study on ESTs which mentioned down-regulations in photosynthesis pathway for the partially resistant cultivar and an up-regulation for the susceptible cultivar [25]. Nevertheless, these two approaches (ESTs analysis and cDNA-AFLP technique) were too different for a complete overlapping of the results, and our different experiments did not underline same aspects of the processes. The ESTs approach was based on large data sets analysis and revealed global patterns, whereas cDNA-AFLP technique allowed fine analysis of genes, even with low expression, and variations of expression can be validated by RT-PCR. Moreover, the experiments were not carried out at the same time, and variations in the conditions of experiments could have thus occurred. A general property of viral infections might cause increasing accumulation of host gene products as part of a stress response, especially HSPs genes. [1]. We observed an up regulation of the expression of HSP70 protein (gb|DQ883891) in wounded leaves and virus infected Azucena leaves harvested at 3 dpi. Many viruses elicit the expression of HSP70 genes (and other heat shock genes) and in some case HSPs have been shown to facilitate viral infection [31]. It seems that most viruses trigger these generic stresslike or defence responses which occur in the absence of typical gene-for-gene or resistance gene-avirulence gene interactions. RYMV infection also induced the expression of WRKY DNA binding protein (gb|DQ884128) in virus infected IR64 leaves at 7dpi. WRKY6, a member of the WRKY family, has been identified by Whitham as elicited by viruses and other pathogen infections [1]. The RYMV caused both specific and general changes in host gene expression and such examples show that this virus elicits expression of common genes induced by other viruses.

This study is the first compiled map on RYMV/Rice interaction. We physically mapped 281 RSR genes using Sim4 [32] on the rice physical map, with respect to intron/exon structure. The map is available, (see Availability and requirements section). We also mapped the ESTs and proteins previously identified [24,25] and the position of the 7 QTLs involved in the partial resistance [7]. The ESTs or cDNA-AFLP analysis revealed changes in host gene expression as a consequence of the infection, whereas the QTLs are involved in a direct response of the tolerance of the host. We did not expect to find any adding up, as the ESTs analysis revealed largely expressed genes, and cDNA-AFLP technique showed up some genes with weak expression level, moreover we do not know if the QTLs involved in partially resistance or tolerance are induced or repressed.

The statistical analysis of the distribution of the RSR sequences showed non uniform distribution of RSR sequences on chromosomes 4 and 10 and thus might underline the hypothesis of RSR sequences clusters of deregulation. We did not observe any cluster regrouping RSR sequences with a similar function ("functional cluster"). But we did not identify all the genes involved in the response to RYMV infection and a deeper study could identify more clusters (especially functional ones) at a microlevel.

Some sequences from IR64 and Azucena colocalized (Table 2). The pattern of gene expression under RYMV infection has been confirmed by quantitative RT-PCR for some RSR genes and showed some differences between the two cultivars. Such RSR genes might be involved in the specificity of the host response. One of these genes encodes an ABC transporter (DQ883962 and DQ884118 RSR sequences). This gene is highly repressed at 5 dpi in the susceptible cultivar and over expressed in wounded leaves of the partially resistant cultivar (Figure 4, A2). ABC transporter genes were the most predominant genes identified by cDNA-AFLP (6 RSR sequences for Azucena and 4 RSR sequences for IR64) and thus might play an important role in plant response to RYMV infection. Such transporters are involved in the membrane transport of a wide range of structurally and functionally unrelated compounds, such as glutathione conjugates, lipids, inorganic acids, peptides, secondary metabolism, toxin and drugs [33]. It has been shown that some ABC transporter, like the AtPDR8, could be a key factor controlling the extent of cell death in the defence response [34], and conferring heavy metal resistance [35]. We also observed a variation of gene expression of a sphingosine-1-phosphate lyase in the two cultivars (Table 2, DQ883945 and DQ884107 RSR sequences). We observed an important decrease of gene expression in RYMV infected leaves of the susceptible cultivar as soon as 2 dpi (Figure 4, A14). Lipids influence pathogenesis and resistance mechanisms associated with plant-microbe interactions, and sphingolipids act as elicitors of defence response [36]. Such families of genes with a down regulation of expression during the kinetic might be targets of small RNAs and deeper study should be undertaken to analyze the potential effects of small RNAs on their regulation of expression. Finally, we noticed the down regulation of 5 different RSR sequences encoding retroelements (DQ884156, DQ883989, DQ883835, DQ883911, and DQ883916). This negative regulation occurred during the whole kinetic in Azucena infected leaves. A deeper transcriptomic analysis is now carried on to determine the relation between the virus infection and such down regulation of retroelement.

## Conclusion

This study presents for the first time a compiled physical map on rice/virus interaction. It gives a strong basis for future studies on RYMV/Rice interaction and for other rice pathogens, as CMAP tool allows the combination of maps. We identified variations of host responses to RYMV infection in susceptible and partially resistant cultivars and we showed that the phenomenon of tolerance to the RYMV involves regulations at different cellular levels. Some genes appeared to be potentially important during the response of partially resistant and susceptible cultivars and it would be interesting to study the variation of their expression in a near isogenic line with only the major QTLs of resistance, this would allow avoiding any bias due to the difference of the genetic background of the 2 cultivars. Moreover, it could be interesting to analyze the variation of expression (using quantitative RT-PCR) of such genes in *O. glaberrima *species with different levels of resistance assuming that the sequencing is available. This would free further studies from inter-species variations of response to virus infection.

## Methods

### Plant materials

Two cultivars were used: IR64 (*O. s. indica*) and Azucena (*O. s. japonica*). IR64 is a high-yielding cultivar developed at the International Rice Research Institute (IRRI) and Azucena is a traditional upland cultivar from the Philippines. Seeds of both cultivars were sown individually and 60 plants were grown in a growth chamber with 12 h of light at 28°C and 12 h of dark at 24°C, 70% relative humidity. For each cultivar, 20 two-leaf stage plants were inoculated with buffer (20 mM phosphate buffer, pH 7), or purified RYMV particles at a concentration of 100 μg/ml. This experiment was repeated twice, in the same conditions. Carborundum was added to the buffer in order to facilitate mechanical inoculation of the leaves. All the plants were grown together and were harvested at the same time in order to minimize potential problems due to factors such as circadian rhythm or harvest time in relation to illumination time. Non-stressed, wounded (buffer inoculated) and RYMV inoculated leaves were harvested 2, 5 and 7 days post inoculation (dpi) for IR64 leaves and 3, 5, 7 dpi for Azucena leaves. These harvest dates were chosen as appropriate times for a well established viral multiplication and corresponded to an equivalent virus accumulation in both cultivars based on RT-PCR analysis (Figure 1).

### RNA extraction and ds-cDNA synthesis

Total RNA was extracted using Qiagen RNeasy Plant mini kit (Qiagen, Germany) and poly A^+ ^RNA was purified from 200 μg of total RNA using Qiagen Oligotex mRNA purification kit (Qiagen, Germany) according to the manufacturer's instructions. The amount and quality of total RNA, poly A^+ ^RNA were checked on 1% agarose non-denaturing gel and by UV illumination. Double-stranded cDNA was synthesized from 3 μg of poly A+ RNA using Stratagene cDNA synthesis kit (Stratagene Inc., La Jolla, CA) according to the manufacturer's instructions.

RYMV accumulation in rice was investigated by RT-PCR following the method described by [37] and according to the manufacturer's instruction (Life Technology). 439 bp fragment was amplified using primers R18: 5'-GGTGTCAGCATAGTCGTAGAG-3' (3839-3819) and R17: 5'-CACACGTGCGGGGTGTGGAG-3' (3380-3400) [38].

### cDNA-AFLP procedure

cDNA-AFLP was performed using RNA extracted from non-stressed and wounded leaves harvested at the first time point (2 dpi for IR64 and 3 dpi for Azucena) and RYMV-infected leaves harvested at 2, 5, and 7 dpi for IR64 and 3, 5, and 7 dpi for Azucena. One microgram of each double strand cDNA sample was digested with 5 U of Eco RI and Mse I (Gibco Invitrogen, Cergy Pontoise, France) and 266 ng of Eco RI- and Mse I-adapters were ligated onto digested cDNA ends. The sequences of adapters were as follow: Eco RI-adapter top strand, 5'-CTCGTAGACTGCGTACC-3'; Eco RI-adapter bottom strand, 5'-AATTGGTACGCAGTC-3'; Mse I-adapter top strand, 5'-GACGATGAGTCCTGAG-3'; Mse I-adapter bottom strand, 5'-TACTCAGGACTCAT-3'. The core sequences of primers for pre-amplification and selective amplification were as follow: Eco RI primer, 5'-GACTGCGTACCAATTC-3'; Mse I primer, 5'-GATGAGTCCTGAGTAA-3'. Selective nucleotides were added to the 3'-end of each primer as follow: zero nucleotide for pre-amplification and two nucleotides (A/T/G/C and A/T/G/C) for selective amplification (256 different primer combinations). The 5'-end of Eco RI primers for selective amplification was radiolabelled using [γ-^33^P]ATP (Amersham Pharmacia Biotech, UK) and T4 polynucleotide kinase (Appligene, Pleasanton, CA) according to the manufacturer's instructions. Pre-amplification and selective amplification were carried out in 50 μl final volume, which contained 20 ng of ligated ds-DNA, 75 ng of both Eco RI and Mse I primers, dNTPs in final concentration of 200 μM, 5 μl of 10 × Promega buffer (Promega, Madison, USA), MgCl_2 _in final concentration of 2.5 mM, and 1 U Taq polymerase (Promega, Madison, USA). PCR was performed on a PTC-200™ thermocycler using the following cycling conditions: initial denaturation at 94°C (30 s); 13 "touchdown" cycles: 0.7°C drop per cycle to a final annealing temperature of 56°C (30 s), 72°C (1 min); followed by 33 cycles: 94°C (30 s); 56°C (30 s); 72°C (1 min). The resulting PCR products were separated by polyacrylamide gel electrophoresis under denaturing conditions, and then the gels were dried on 3 mm (Whatman, Maidstone, UK) paper and exposed to Biomax-MS X-ray film (Kodak).

### Isolation and sequencing of amplified cDNA products

The bands of interest were marked on the films, excised from the gels and placed in sterile tubes. DNA fragments were extracted from the denaturing gel by immerging the gel bands in 50 μl of sterile water, and allowed to stand overnight at +4°C in order to allow the DNA fragments to diffuse. After centrifugation, the extracts were re-amplified using AFLP selective primers under the same PCR conditions as AFLP. The DNA fragments were then cloned into pGEM^®^-T Easy vector (Promega, Madison, USA) according to manufacturer's instructions. Three individual clones were isolated and maintained for each AFLP band.

### Sequencing and analysis

Plasmid DNA was prepared using Qiagen R.E.A.L. kit. Sequencing reactions were carried out with Applied Biosystems BigDye terminator kits and analyzed on an Applied Biosystems (Courtaboeuf France) 3100 sequencer. The nucleotide sequences were analyzed for homology against GenBank non-redundant database and rice ESTs using the Basic Local Alignment Search Tool (BLAST) program [39].

### Quantitative PCR

We ran two independent sets of experiments comprising 10 healthy plants, 10 wounded plants, and 10 plants infected by the RYMV. Total RNA were extracted from 14 days-old leaves harvested at 2 and 5 days post inoculation. We used RNeasy kit (Qiagen). Poly(dT) cDNA was prepared out of three times 400 ng total RNA using Superscript III (Invitrogen). Quantification was performed on a Stratagene Mx3005P apparatus with the FullVelocity SYBR Green QPCR Master Mix (Stratagene) upon recommendations of the manufacturer. PCR was carried out in 96-well optical reaction plates heated for 5 minutes to 95°C, followed by 40 cycles of 10 seconds at 95°C and annealing-extension for 30 seconds at 60°C. Target quantifications were performed with specific primer pairs designed using Beacon Designer 4.0 (Premier Biosoft International, Palo Alto, USA). Expression levels were normalized to *ACTIN2 *(At3g18780). For each experiment, all the RT-PCR was performed in triplicates and the presented values represent means plus/minus standard deviation.

### Mapping

This step was not entirely straightforward since we aligned our cDNA to genomic sequences. We used the fifth release of the 12 rice chromosomes [21]. We used SIM4 for alignment comparison which considers intron/exon structure and can map expressed sequence tags (EST) or cDNA sequence to a genome. Because the cDNA sequences are derived from two *Oryza sativa *ecotypes (IR64, *O. s. indica *and Azucena, *O. s. japonica*) that differ from the genome's ecotype (Nipponbare, *O. s. japonica*), we expected to find some minor polymorphisms when we compared the IR64 cDNAs to the genome sequence of Nipponbare. Some molecular analyses have consistently shown a difference between *indica *and *japonica *in the quantification of genomic DNA and repetitive sequence [40]. Nevertheless, a recent comparative approach on fine physical map of chromosome 4 [41] reveals that the *indica *and *japonica *physical maps showed an overall synteny even with some intraspecific DNA-sequence polymorphisms: insertion/deletion (indel) and single nucleotide polymorphism (SNP) [16]. A comparative genomics program entitled "*Oryza *Map Alignment Project" (OMAP) has been started to study evolution, genome organization, domestication, gene regulatory network, and crop improvement [42,43]. Moreover, the average of numbers of introns and exons per gene, gene content, and order are highly conserved in *indica *and *japonica *sequences [44], and thus it appears reasonable to use the Nipponbare sequence as a reference to map and analyse rice gene sequences.

A first filter was the global percentage of identity: all RSR sequences with more than 80% of identity were selected. All RSR sequences with less than 98% of homology for more than 50% of the sequence were deleted. Then, all sequences with more than 95% of identity for each exon (except the last one which may contain large number of N) were kept. Thus, these sequences were mapped onto the chromosomes and referenced by the position of the first exon.

We also mapped the ESTs previously identified as involved in this interaction rice/RYMV [25] using the same approach. The proteins shown as involved in the interaction were positioned using a blastx subroutine to compare the sequence of each peptide to the chromosome [24]. These ESTs and proteins are mentioned on the right of the map.

### Statistical analysis

The distribution of RSR sequences per megabase was compared to the distribution of ATG observed per megabase along the chromosomes using the sub-program STRUC from GENEPOP version 3.1c [45]. This program uses Markov chain method to estimate without bias the exact *P*-value of the probability test (or Fisher exact test) on contingency tables of any sizes. The contingency test was performed for each chromosome with an alpha error < 0.05.

## Availability and requirements

: Physical map of 281 RSR sequences. On the left of each chromosome, start positions of RSR sequences were in bp *100000, and the ESTs and proteins identified as candidates involved in host response [24,25]. On the left, we mentioned the position of the 7 QTLs involved in the partial resistance [7].

## Authors' contributions

MVD and CB both designed and coordinated the study. MVD carried out the molecular genetic work and the statistical analyses, participated to the annotation and the mapping, and drafted the manuscript. CTD carried out the bioinformatics and the physical mapping. TTHN participated to the cDNA-AFLP assays. MB and CS carried out the quantitative RT-PCR. MD participated in the coordination of the study and helped to draft the manuscript. All authors read and approved the final manuscript.

## Supplementary Material

Additional file 1 Table of RSR sequences classified into functional categories. The table provided represents the classification of all RSR sequences with a putative function. The RSR sequences were blasted against the NR database (All non-redundant GenBank CDS translations + RefSeq Proteins + PDB + SwissProt + PIR + PRF). Click here for file
