# Supplementary material for: Rice Yellow Mottle Virus stress responsive genes from susceptible and tolerant rice genotypes
Source: BMC Plant Biol. 2008 Mar 3;8:26. doi: 10.1186/1471-2229-8-26 (PMC2275266; doi:10.1186/1471-2229-8-26)
Supplement: Additional file 1 — Table of RSR sequences classified into functional categories. The table provided represents the classification of all RSR sequences with a putative function. The RSR sequences were blasted against the NR database (All non-redundant GenBank CDS translations + RefSeq Proteins + PDB + SwissProt + PIR + PRF). [file 1471-2229-8-26-S1.doc]

Additional Table. Functional class analysis of the identified sequences

a: cDNA-AFLP fragment GenBank accession. b: pattern of deregulation, inside brackets BI for buffer inoculated control or *n* dpi (*n* days post inoculation), + for up regulation, - for down regulation. c: putative function as found after blasting against NR database.

|  | **GB Id^a^** | **Deregulation^b^** | **Description^c^** | **Organism** | **E value** | **% identity** |
| --- | --- | --- | --- | --- | --- | --- |
| **AZUCENA** |  |  |  |  |  |  |
| Defense |  |  |  |  |  |  |
|  | gb|DQ883904 | (7dpi)+ | emb|CAA81059.1| salT | Oryza sativa | 2.00E-09 | 100 |
|  | gb|DQ883891 | (BI,3dpi)+ | ref|NP_910052.1| Hsp70 binding protein | Oryza sativa | 3.00E-50 | 89 |
|  | gb|DQ883879 | (5,7dpi)- | dbj|BAD94167.1| multi-drug resistance protein | Arabidopsis thaliana | 9.00E-22 | 85 |
|  | gb|DQ883862 | (7dpi)+ | emb|CAA81059.1| salT | Oryza sativa | 2.00E-08 | 97 |
|  | gb|DQ883975 | (7dpi)+ | emb|CAA81059.1| salT | Oryza sativa | 2.00E-09 | 100 |
|  | gb|DQ883943 | (BI,3dpi)+ | ref|NP_910308.1| chaperonin 60 beta precursor | Oryza sativa | 1.00E-11 | 71 |
|  | gb|DQ883924 | (5,7dpi)+ | gb|AAX94877.1| Similar to NBS-LRR disease resistance protein homologue | Oryza sativa | 5.00E-15 | 91 |
| Cellular Organisation | |  |  |  |  |  |
|  | gb|DQ883992 | (5,7dpi)- | ref|XP_419249.1| MGC53359 protein | Gallus gallus | 4.00E-29 | 100 |
|  | gb|DQ883838 | (5dpi)+ | dbj|BAD35845.1| kinesin motor protein-related-like | Oryza sativa | 2.00E-12 | 100 |
|  | gb|DQ883971 | (5,7dpi)- | gb|AAH83344.1| Tubulin, alpha 1 | Mus musculus | 8.00E-29 | 100 |
| Energy |  |  |  |  |  |  |
|  | gb|DQ883984 | (5dpi)+ | dbj|BAD27711.1| Citrate synthase, glyoxysomal precursor (GCS) | Oryza sativa | 2.00E-05 | 100 |
| Metabolism | |  |  |  |  |  |
|  | gb|DQ883892 | (3dpi)+ | ref|XP_467290.1| 4-coumarate:CoA ligase isoform 2 | Oryza sativa | 6.00E-07 | 97 |
|  | gb|DQ883886 | (BI)+ | emb|CAA33911.1| NADH dehydrogenase 49kDa protein | Oryza sativa | 6.00E-43 | 99 |
|  | gb|DQ883844 | (3,5,7dpi)- | gb|AAS46130.1| cytochrome f; petA | Oryza sativa | 9.00E-25 | 100 |
|  | gb|DQ884010 | (BI,3,5,7dpi)- | dbj|BAD72330.1| acyl-CoA synthetase | Oryza sativa | 2.00E-17 | 98 |
|  | gb|DQ883906 | (3,5,7dpi)- | gb|AAK00824.1| NADH dehydrogenase subunit F | Leptonychia | 1.00E-39 | 79 |
|  | gb|DQ883907 | (5,7dpi)+ | ref|NP_922817.1| pyruvate kinase | Oryza sativa | 1.00E-47 | 100 |
|  | gb|DQ883995 | (BI,3,5,7dpi)- | ref|NP_914907.1| beta-glucosidase | Oryza sativa | 5.00E-20 | 73 |
|  | gb|DQ883990 | (BI,3,7dpi)- | gb|AAT85082.1| pyruvate orthophosphate dikinase | Oryza sativa | 1.00E-38 | 100 |
|  | gb|DQ883959 | (BI,3,5,7dpi)- | ref|NP_909094.1| mannose-6-phosphate isomerase | Oryza sativa | 2.00E-29 | 79 |
| Photosynthesis | |  |  |  |  |  |
|  | gb|DQ884058 | (5,7dpi)- | gb|AAS46139.1| photosystem II phosphoprotein; psbH | Oryza sativa | 6.00E-07 | 100 |
| Protein Biosynthesis | |  |  |  |  |  |
|  | gb|DQ883920 | (7dpi)+ | ref|NP_915777.1| subtilase | Oryza sativa | 2.00E-18 | 98 |
|  | gb|DQ883915 | (5,7dpi)- | ref|XP_470568.1| phytoene dehydrogenase precursor | Oryza sativa | 2.00E-15 | 95 |
|  | gb|DQ883912 | (5,7dpi)+ | ref|NP_916943.1| beclin 1 | Oryza sativa | 8.00E-23 | 81 |
|  | gb|DQ883901 | (3,5,7dpi)- | emb|CAA55027.1| large ribosomal protein 23 | Hordeum vulgare | 1.00E-04 | 100 |
|  | gb|DQ883900 | (BI,3dpi)- | emb|CAA55027.1| large ribosomal protein 23 | Hordeum vulgare | 1.00E-04 | 100 |
|  | gb|DQ883876 | (2,5,7dpi)- | gb|ABA96309.1| Clp amino terminal domain, putative | Oryza sativa | 1.00E-33 | 97 |
|  | gb|DQ884147 | (7dpi)+ | ref|XP_467672.1| dihydropyrimidine dehydrogenase | Oryza sativa | 5.00E-12 | 100 |
|  | gb|DQ884145 | (5dpi)+ | dbj|BAD29529.1| F-box protein-like | Oryza sativa | 7.00E-11 | 97 |
|  | gb|DQ883848 | (BI,3dpi)+ | ref|NP_909852.1| thiamin biosynthesis protein | Oryza sativa | 2.00E-25 | 96 |
|  | gb|DQ883841 | (BI,5 dpi)+ | gb|AAD02267.1| ClpC protease | Spinacia oleracea | 1.00E-14 | 100 |
|  | gb|DQ884004 | (5,7dpi)- | gb|AAT93972.1| DegP2 protease | Oryza sativa | 1.00E-40 | 100 |
|  | gb|DQ884002 | (BI,3,5,7dpi)- | dbj|BAD82722.1| ribosomal protein-like | Oryza sativa | 7.00E-09 | 97 |
|  | gb|DQ883999 | (BI,3,5,7dpi)- | ref|XP_469565.1| splicing factor | Oryza sativa | 3.00E-06 | 100 |
|  | gb|DQ883976 | (3dpi)+ | ref|NP_921725.1| acetohydroxyacid isomeroreductase | Oryza sativa | 3.00E-11 | 97 |
|  | gb|DQ883944 | (BI,3dpi)- | sp|Q9LSU1|PSA5_ORYSA Proteasome subunit alpha type 5 | Oryza sativa | 5.00E-14 | 88 |
| Signal transduction | |  |  |  |  |  |
|  | gb|DQ883917 | (5,7dpi)- | ref|NP_849466.1| kinase/ ribokinase | Arabidopsis thaliana | 2.00E-36 | 66 |
|  | gb|DQ883902 | (5dpi)+ | ref|XP_466203.1| MEK kinase | Oryza sativa | 4.00E-13 | 100 |
|  | gb|DQ883867 | (BI,3,5,7dpi)- | ref|NP_908964.1| receptor serine/threonine kinase PR5K | Oryza sativa | 3.00E-18 | 100 |
|  | gb|DQ884153 | (5,7dpi)+ | gb|AAG43286.2| auxin response factor 1 | Oryza sativa | 4.00E-20 | 100 |
|  | gb|DQ884020 | (5,7dpi)- | ref|XP_450601.1| serine/threonine protein kinase | Oryza sativa | 4.00E-20 | 96 |
|  | gb|DQ884007 | (BI,3dpi)- | ref|NP_910025.1| receptor kinase | Oryza sativa | 2.00E-10 | 100 |
|  | gb|DQ884006 | (5dpi)+ | ref|NP_910025.1| receptor kinase | Oryza sativa | 7.00E-06 | 83 |
|  | gb|DQ884005 | (BI,3,5,7dpi)+ | ref|NP_910025.1| receptor kinase | Oryza sativa | 5.00E-11 | 100 |
|  | gb|DQ884000 | (5,7dpi)- | ref|NP_912467.1| serine/threonine protein kinase | Oryza sativa | 2.00E-10 | 100 |
|  | gb|DQ883970 | (5,7dpi)+ | ref|NP_912826.1| protein kinase | Oryza sativa | 5.00E-20 | 98 |
|  | gb|DQ883952 | (BI)+ | gb|AAT77080.1| inositol phosphate kinase | Oryza sativa | 3.00E-28 | 97 |
|  | gb|DQ883950 | (3,5,7dpi)+ | gb|AAT77080.1| inositol phosphate kinase | Oryza sativa | 6.00E-29 | 98 |
|  | gb|DQ883945 | (7dpi)+ | gb|AAX89367.1| sphingosine-1-phosphate lyase | Oryza sativa | 1.00E-04 | 100 |
| Transcription | |  |  |  |  |  |
|  | gb|DQ883919 | (7dpi)+ | gb|AAU10677.1| G-box binding factor | Oryza sativa | 5.00E-12 | 97 |
|  | gb|DQ883897 | (7dpi)+ | dbj|BAD82793.1| ABC1-like | Oryza sativa | 2.00E-31 | 99 |
|  | gb|DQ883825 | (3dpi)+ | ref|XP_463980.1| transcription factor HBP-1a | Oryza sativa | 7.00E-30 | 96 |
|  | gb|DQ884013 | (5dpi)+ | ref|XP_469113.1| DNA-binding protein | Oryza sativa | 2.00E-22 | 100 |
|  | gb|DQ883986 | (3dpi)+ | ref|XP_468125.1| transcription factor EREBP1 | Oryza sativa | 4.00E-33 | 100 |
|  | gb|DQ883981 | (BI)+ | ref|XP_468125.1| transcription factor EREBP1 | Oryza sativa | 4.00E-33 | 100 |
|  | gb|DQ883980 | (BI)+ | ref|XP_468125.1| transcription factor EREBP1 | Oryza sativa | 4.00E-33 | 100 |
|  | gb|DQ883969 | (3dpi)+ | ref|NP_919608.1| DNA2-NAM7 helicase family protein | Oryza sativa | 8.00E-23 | 80 |
| Transport |  |  |  |  |  |  |
|  | gb|DQ883875 | (7dpi)+ | ref|XP_480212.1| WD-40 repeat protein-like | Oryza sativa | 2.00E-06 | 96 |
|  | gb|DQ883870 | (BI,3dpi)+ | dbj|BAD29207.1| PDR-type ABC transporter-like | Oryza sativa | 4.00E-16 | 100 |
|  | gb|DQ883864 | (BI,3dpi)- | emb|CAD20998.1| potassium transporter | Oryza sativa | 1.00E-34 | 100 |
|  | gb|DQ884154 | (BI,3dpi)- | ref|XP_550309.1| band 3 anion transport protein -like | Oryza sativa | 1.00E-12 | 100 |
|  | gb|DQ884056 | (5,7dpi)+ | ref|XP_469514.1| alpha-coat protein | Oryza sativa | 3.00E-07 | 100 |
|  | gb|DQ884017 | (7dpi)+ | gb|AAK53758.1| potassium transporter HAK1p | Mesembryanthemum crystallinum | 4.00E-05 | 100 |
|  | gb|DQ884016 | (5,7dpi)- | gb|AAK53758.1| potassium transporter HAK1p | Mesembryanthemum crystallinum | 4.00E-05 | 100 |
|  | gb|DQ883994 | (7dpi)- | gb|ABA91479.1| Sodium/calcium exchanger protein, putative | Oryza sativa | 1.00E-67 | 98 |
|  | gb|DQ883977 | (5,7dpi)+ | ref|XP_480212.1| WD-40 repeat protein-like | Oryza sativa | 3.00E-06 | 100 |
|  | gb|DQ883963 | (5,7dpi)+ | ref|NP_915325.1| ABC transporter | Oryza sativa | 2.00E-42 | 48 |
|  | gb|DQ883962 | (BI+) | ref|NP_915325.1| ABC transporter | Oryza sativa | 2.00E-57 | 98 |
|  | gb|DQ883935 | (5dpi)+ | dbj|BAD27735.1| peroxisomal targeting signal type 2 receptor | Oryza sativa | 1.00E-12 | 100 |
|  | gb|DQ883933 | (5,7dpi)- | dbj|BAD27735.1| peroxisomal targeting signal type 2 receptor | Oryza sativa | 2.00E-11 | 97 |
| Retrotransposons | |  |  |  |  |  |
|  | gb|DQ883887 | (5,7dpi)- | ref|NP_918586.1| OsTATC | Oryza sativa | 2.00E-20 | 100 |
| Unclassified | |  |  |  |  |  |
|  | gb|DQ883918 | (5,7dpi)- | ref|NP_921527.1| unknown protein | Oryza sativa | 3.00E-18 | 21 |
|  | gb|DQ883916 | (3,5,7dpi)- | ref|XP_471724.1| OSJNBa0023J03.1 | Oryza sativa | 1.00E-08 | 46 |
|  | gb|DQ883911 | (3,5,7dpi)- | gb|AAT85026.1| hypothetical protein | Oryza sativa | 7.00E-33 | 72 |
|  | gb|DQ883910 | (5dpi)+ | ref|XP_474354.1| OSJNBa0064G10.19 | Oryza sativa | 2.00E-07 | 100 |
|  | gb|DQ883905 | (7dpi)+ | dbj|BAD32899.1| membrane protein-like | Oryza sativa | 3.00E-13 | 100 |
|  | gb|DQ883895 | (5dpi)+ | ref|XP_473410.1| OSJNBb0034G17.1 | Oryza sativa | 1.00E-07 | 100 |
|  | gb|DQ883894 | (3,5,7dpi)+ | ref|XP_471720.1| OSJNBa0079M09.13 | Oryza sativa | 2.00E-21 | 100 |
|  | gb|DQ883893 | (7dpi)+ | ref|XP_471720.1| OSJNBa0079M09.13 | Oryza sativa | 2.00E-21 | 100 |
|  | gb|DQ883828 | (5,7dpi)+ | gb|ABA91300.1| expressed protein | Oryza sativa | 3.00E-17 | 100 |
|  | gb|DQ883871 | (3,5,7dpi)- | gb|AAV24913.1| polyprotein | Oryza sativa | 4.00E-08 | 100 |
|  | gb|DQ883869 | (7dpi)+ | ref|XP_472222.1| OSJNBa0083D01.23 | Oryza sativa | 1.00E-09 | 80 |
|  | gb|DQ883865 | (5,7dpi)+ | gb|ABA99008.1| expressed protein | Oryza sativa | 1.00E-12 | 100 |
|  | gb|DQ883861 | (5,7dpi)- | ref|XP_549856.1| unknown protein | Oryza sativa | 2.00E-11 | 100 |
|  | gb|DQ884159 | (BI,3,5,7dpi)- | gb|AAT77332.1| unknown protein | Oryza sativa | 4.00E-14 | 34 |
|  | gb|DQ884156 | (3,5,7dpi)- | ref|NP_908696.1| OSJNBa0011P19.23 | Oryza sativa | 8.00E-05 | 91 |
|  | gb|DQ884140 | (3,5,7dpi)- | ref|XP_469793.1| unknown protein | Oryza sativa | 2.00E-05 | 88 |
|  | gb|DQ883852 | (BI,3,5dpi)+ | gb|AAP45177.1| hypothetical protein 177O13.35 | Solanum bulbocastanum | 5.00E-06 | 86 |
|  | gb|DQ883850 | (7dpi)+ | gb|AAO61994.1| conserved hypothetical protein | Aster yellows phytoplasma | 7.00E-09 | 75 |
|  | gb|DQ883843 | (5,7dpi)+ | emb|CAB79871.1| protein | Arabidopsis thaliana | 7.00E-06 | 83 |
|  | gb|DQ884019 | (5,7dpi)- | ref|XP_471020.1| OSJNBb0060M15.10 | Oryza sativa | 3.00E-07 | 74 |
|  | gb|DQ884014 | (BI,3dpi)+ | ref|XP_467132.1| unknown protein | Oryza sativa | 2.00E-05 | 96 |
|  | gb|DQ884011 | (Bi,3,5,7dpi)- | gb|ABA94690.1| expressed protein | Oryza sativa | 2.00E-08 | 100 |
|  | gb|DQ883996 | (5,7dpi)+ | ref|XP_464418.1| hypothetical protein | Oryza sativa | 4.00E-07 | 83 |
|  | gb|DQ883993 | (7dpi)+ | ref|XP_470997.1| OSJNBa0027H06.5 | Oryza sativa | 1.00E-49 | 97 |
|  | gb|DQ883991 | (3dpi)+ | ref|XP_481014.1| ORF249 | Oryza sativa | 4.00E-09 | 97 |
|  | gb|DQ883989 | (3,5,7dpi)- | ref|XP_476190.1| unknown protein | Oryza sativa | 5.00E-35 | 96 |
|  | gb|DQ883837 | (BI,3dpi)+ | ref|XP_476011.1| unknown protein | Oryza sativa | 1.00E-15 | 94 |
|  | gb|DQ883973 | (BI,3dpi)+ | ref|XP_506339.1| PREDICTED OJ1136_F08.109 gene product | Oryza sativa | 6.00E-07 | 72 |
|  | gb|DQ883972 | (7dpi)+ | dbj|BAA22288.1| polyprotein | Oryza australiensis | 5.00E-20 | 65 |
|  | gb|DQ883966 | (5,7dpi)- | ref|NP_915543.1| P0529E05.22 | Oryza sativa | 9.00E-06 | 77 |
|  | gb|DQ883835 | (3,5,7dpi)- | gb|AAV31373.1| polyprotein | Oryza sativa | 9.00E-05 | 100 |
|  | gb|DQ883957 | (3,5,7dpi)- | ref|XP_464456.1| hypothetical protein | Oryza sativa | 7.00E-08 | 67 |
|  | gb|DQ883834 | (7dpi)+ | ref|XP_473337.1| OSJNBa0091D06.15 | Oryza sativa | 1.00E-16 | 100 |
|  | gb|DQ883941 | (5dpi)+ | emb|CAE05443.2| OSJNBa0073E02.3 | Oryza sativa | 2.00E-17 | 100 |
|  | gb|DQ883932 | (BI,3dpi)- | ref|XP_469441.1| expressed protein | Oryza sativa | 8.00E-06 | 100 |
|  | gb|DQ883926 | (5,7dpi)+ | ref|XP_471556.1| OSJNBa0019J05.17 | Oryza sativa | 1.00E-15 | 100 |
|  | gb|DQ883925 | (5dpi)+ | ref|XP_478635.1| pentatricopeptide (PPR) repeat-containing protein-like protein | Oryza sativa | 4.00E-05 | 100 |
|  | gb|DQ883921 | (7dpi)+ | ref|XP_473940.1| OSJNBb0085C12.17 | Oryza sativa | 9.00E-13 | 98 |
|  | gb|DQ883914 | (BI,3dpi)+ | - | - | - | - |
|  | gb|DQ883913 | (5dpi)+ | - | - | - | - |
|  | gb|DQ883830 | (BI)+,(7dpi)- | - | - | - | - |
|  | gb|DQ883909 | (5,7dpi)+ | - | - | - | - |
|  | gb|DQ883908 | (5dpi)+ | - | - | - | - |
|  | gb|DQ883903 | (7dpi)+ | - | - | - | - |
|  | gb|DQ883829 | (5,7dpi)+ | - | - | - | - |
|  | gb|DQ883899 | (Bi)+ | - | - | - | - |
|  | gb|DQ883898 | (5dpi)+ | - | - | - | - |
|  | gb|DQ883896 | (7dpi)+ | - | - | - | - |
|  | gb|DQ883890 | (5,7dpi)+ | - | - | - | - |
|  | gb|DQ883889 | (BI,3dpi)+ | - | - | - | - |
|  | gb|DQ883888 | (BI,3dpi)- | - | - | - | - |
|  | gb|DQ883885 | (5,7dpi)+ | - | - | - | - |
|  | gb|DQ883884 | (7dpi)+ | - | - | - | - |
|  | gb|DQ883883 | (Bi)+ | - | - | - | - |
|  | gb|DQ883882 | (Bi)+ | - | - | - | - |
|  | gb|DQ883881 | (BI)+ | - | - | - | - |
|  | gb|DQ883827 | (5,7dpi)- | - | - | - | - |
|  | gb|DQ883880 | (BI,3,5,7dpi)+ | - | - | - | - |
|  | gb|DQ883878 | (5,7dpi)+ | - | - | - | - |
|  | gb|DQ883877 | (5dpi)+ | - | - | - | - |
|  | gb|DQ883874 | (5,7dpi)+ | - | - | - | - |
|  | gb|DQ883873 | (7dpi)+ | - | - | - | - |
|  | gb|DQ883872 | (BI,3dpi)- | - | - | - | - |
|  | gb|DQ883826 | (BI,3,5,7dpi)- | - | - | - | - |
|  | gb|DQ883868 | (7dpi)+ | - | - | - | - |
|  | gb|DQ883866 | (3,5,7dpi)- | - | - | - | - |
|  | gb|DQ883863 | (5,7dpi)+ | - | - | - | - |
|  | gb|DQ883860 | (BI,3,5,7dpi)- | - | - | - | - |
|  | gb|DQ883859 | (BI,3,5dpi)- | - | - | - | - |
|  | gb|DQ883858 | (3,5,7dpi)- | - | - | - | - |
|  | gb|DQ883857 | (3,5,7dpi)+ | - | - | - | - |
|  | gb|DQ883856 | (BI,3,5,7dpi)+ | - | - | - | - |
|  | gb|DQ883855 | (BI,3,5,7dpi)- | - | - | - | - |
|  | gb|DQ884158 | (3dpi)+ | - | - | - | - |
|  | gb|DQ884157 | (5,7dpi)+ | - | - | - | - |
|  | gb|DQ884155 | (5dpi)+ | - | - | - | - |
|  | gb|DQ884152 | (BI,3,5,7dpi)- | - | - | - | - |
|  | gb|DQ884151 | (3,5,7dpi)- | - | - | - | - |
|  | gb|DQ884150 | (5,7dpi)+ | - | - | - | - |
|  | gb|DQ883854 | (5,7dpi)- | - | - | - | - |
|  | gb|DQ884149 | (5,7dpi)- | - | - | - | - |
|  | gb|DQ884148 | (3dpi)+ | - | - | - | - |
|  | gb|DQ884146 | (5,7dpi)+ | - | - | - | - |
|  | gb|DQ884144 | (BI+) | - | - | - | - |
|  | gb|DQ884143 | (5dpi)+ | - | - | - | - |
|  | gb|DQ884142 | (7dpi)+ | - | - | - | - |
|  | gb|DQ884141 | (7dpi)+ | - | - | - | - |
|  | gb|DQ883853 | (5,7dpi)- | - | - | - | - |
|  | gb|DQ884139 | (BI)+ | - | - | - | - |
|  | gb|DQ883851 | (5,7dpi)+ | - | - | - | - |
|  | gb|DQ883849 | (5,7dpi)+ | - | - | - | - |
|  | gb|DQ883847 | (5,7dpi)+ | - | - | - | - |
|  | gb|DQ883846 | (5,7dpi)+ | - | - | - | - |
|  | gb|DQ883845 | (5,7dpi)- | - | - | - | - |
|  | gb|DQ884057 | (3dpi)+ | - | - | - | - |
|  | gb|DQ884055 | (5,7dpi)+ | - | - | - | - |
|  | gb|DQ883842 | (5,7dpi)+ | - | - | - | - |
|  | gb|DQ884018 | (5,7dpi)- | - | - | - | - |
|  | gb|DQ884015 | (5,7dpi)+ | - | - | - | - |
|  | gb|DQ884012 | (5dpi)+ | - | - | - | - |
|  | gb|DQ883840 | (3dpi)+ | - | - | - | - |
|  | gb|DQ884009 | (BI,2,5,7dpi)- | - | - | - | - |
|  | gb|DQ884008 | (BI,3dpi)+ | - | - | - | - |
|  | gb|DQ884003 | (BI,3dpi)- | - | - | - | - |
|  | gb|DQ884001 | (5,7dpi)- | - | - | - | - |
|  | gb|DQ883839 | (5dpi)+ | - | - | - | - |
|  | gb|DQ883998 | (BI,3,5,7dpi)+ | - | - | - | - |
|  | gb|DQ883997 | (BI,3,5,7dpi)- | - | - | - | - |
|  | gb|DQ883988 | (3,5,7dpi)- | - | - | - | - |
|  | gb|DQ883987 | (BI)+ | - | - | - | - |
|  | gb|DQ883985 | (BI)- | - | - | - | - |
|  | gb|DQ883983 | (3,5,7dpi)- | - | - | - | - |
|  | gb|DQ883982 | (BI)+ | - | - | - | - |
|  | gb|DQ883979 | (5,7dpi)- | - | - | - | - |
|  | gb|DQ883978 | (3,5,7dpi)- | - | - | - | - |
|  | gb|DQ883974 | (5,7dpi)- | - | - | - | - |
|  | gb|DQ883836 | (BI,2dpi)- | - | - | - | - |
|  | gb|DQ883968 | (5dpi)+ | - | - | - | - |
|  | gb|DQ883967 | (5dpi)+ | - | - | - | - |
|  | gb|DQ883965 | (BI,3,5,7dpi)- | - | - | - | - |
|  | gb|DQ883964 | (BI,3,5,7dpi)- | - | - | - | - |
|  | gb|DQ883961 | (2dpi)+ | - | - | - | - |
|  | gb|DQ883960 | (BI)+ | - | - | - | - |
|  | gb|DQ883958 | (5,7dpi)- | - | - | - | - |
|  | gb|DQ883956 | (BI,3dpi)- | - | - | - | - |
|  | gb|DQ883955 | (BI,3dpi)- | - | - | - | - |
|  | gb|DQ883954 | (BI,3dpi)- | - | - | - | - |
|  | gb|DQ883953 | (5,7dpi)- | - | - | - | - |
|  | gb|DQ883951 | (BI)+ | - | - | - | - |
|  | gb|DQ883949 | (5,7dpi)+ | - | - | - | - |
|  | gb|DQ883948 | (5,7dpi)- | - | - | - | - |
|  | gb|DQ883947 | (BI,3dpi)+ | - | - | - | - |
|  | gb|DQ883946 | (5dpi)+ | - | - | - | - |
|  | gb|DQ883942 | (BI,3,5,7dpi)- | - | - | - | - |
|  | gb|DQ883833 | (7dpi)+ | - | - | - | - |
|  | gb|DQ883940 | (5,7dpi)+ | - | - | - | - |
|  | gb|DQ883939 | (5,7dpi)+ | - | - | - | - |
|  | gb|DQ883938 | (5,7dpi)- | - | - | - | - |
|  | gb|DQ883937 | (5dpi)+ | - | - | - | - |
|  | gb|DQ883936 | (3dpi)+ | - | - | - | - |
|  | gb|DQ883934 | (5,7dpi)- | - | - | - | - |
|  | gb|DQ883931 | (5,7dpi)+ | - | - | - | - |
|  | gb|DQ883832 | (5,7dpi)+ | - | - | - | - |
|  | gb|DQ883930 | (7dpi)+ | - | - | - | - |
|  | gb|DQ883929 | (5dpi)+ | - | - | - | - |
|  | gb|DQ883928 | (5dpi)- | - | - | - | - |
|  | gb|DQ883927 | (5,7dpi)- | - | - | - | - |
|  | gb|DQ883923 | (5,7dpi)- | - | - | - | - |
|  | gb|DQ883922 | (7dpi)+ | - | - | - | - |
|  | gb|DQ883831 | (5,7dpi)+ | - | - | - | - |
|  | gb|DQ883824 | (5,7dpi)+ | - | - | - | - |
| **IR64** |  |  |  |  |  |  |
| Defense |  |  |  |  |  |  |
|  | gb|DQ884090 | (7dpi)+ | ref|XP_470535.1| lipoxygenase | Oryza sativa | 2.00E-10 | 100 |
|  | gb|DQ884063 | (5,7dpi)- | gb|AAV43842.1| DnaJ protein | Oryza sativa | 5.00E-30 | 98 |
|  | gb|DQ884022 | (BI,2,5dpi)- | gb|AAR25995.1| senescence-associated protein | Pyrus communis | 8.00E-13 | 67 |
|  | gb|DQ884021 | (5,7dpi)- | gb|AAR25995.1| senescence-associated protein | Pyrus communis | 8.00E-42 | 98 |
| Cellular Organisation | |  |  |  |  |  |
|  | gb|DQ884073 | (5,7dpi)- | ref|XP_467400.1| kinesin light chain | Oryza sativa | 1.00E-15 | 97 |
| Energy |  |  |  |  |  |  |
|  | gb|DQ884034 | (7dpi)+ | ref|NP_912236.1| ATP synthase delta' chain, mitochondrial precursor | Oryza sativa | 8.00E-10 | 100 |
| Metabolism | |  |  |  |  |  |
|  | gb|DQ884098 | (BI)+ | gb|AAM74942.1| ferritin | Oryza sativa | 4.00E-12 | 100 |
|  | gb|DQ884115 | (5,7dpi)+ | dbj|BAD53501.1| serine carboxylase II-2 | Oryza sativa | 7.00E-06 | 89 |
|  | gb|DQ884104 | (BI,2,5,7dpi)+ | dbj|BAD45927.1| NADH dehydrogenase subunit 4 | Oryza sativa | 9.00E-09 | 94 |
|  | gb|DQ884103 | (BI,2,5,7dpi)+ | dbj|BAD45927.1| NADH dehydrogenase subunit 4 | Oryza sativa | 5.00E-10 | 100 |
|  | gb|DQ884079 | (5dpi)+ | ref|XP_467290.1| 4-coumarate:CoA ligase isoform 2 | Oryza sativa | 1.00E-05 | 90 |
| Photosynthesis | |  |  |  |  |  |
|  | gb|DQ884136 | (BI,2dpi)+ | dbj|BAD62115.1| photosystem II stability/assembly factor HCF136, chloroplast | Oryza sativa | 2.00E-38 | 69 |
|  | gb|DQ884135 | (BI,2,5dpi)+ | dbj|BAD62115.1| photosystem II stability/assembly factor HCF136, chloroplast | Oryza sativa | 7.00E-38 | 69 |
| Protein Biosynthesis | |  |  |  |  |  |
|  | gb|DQ884125 | (5,7dpi)- | ref|XP_464026.1| hosphatidylinositol/phosphatidylcholine transfer protein | Oryza sativa | 1.00E-29 | 97 |
|  | gb|DQ884066 | (BI,2dpi)+ | ref|NP_915866.1| 50S ribosomal protein L34 | Oryza sativa | 2.00E-36 | 99 |
|  | gb|DQ884047 | (7dpi)+ | dbj|BAD52841.1| insulin degrading enzyme | Oryza sativa | 6.00E-07 | 96 |
|  | gb|DQ884035 | (5,7dpi)- | gb|AAT93972.1| DegP2 protease | Oryza sativa | 1.00E-29 | 94 |
|  | gb|DQ884024 | (5,7dpi)+ | ref|XP_465810.1| Ras-GTPase-activating protein binding protein 1 | Oryza sativa | 2.00E-13 | 100 |
|  | gb|DQ884122 | (5,7dpi)- | ref|XP_480160.1| 27k vesicle-associated membrane protein-associated protein | Oryza sativa | 4.00E-36 | 100 |
|  | gb|DQ884113 | (BI)+ | dbj|BAD30375.1| eIF3e | Oryza sativa | 2.00E-28 | 98 |
|  | gb|DQ884111 | (5,7dpi)- | dbj|BAD67983.1| GOS9 | Oryza sativa | 5.00E-20 | 81 |
| Signal transduction | |  |  |  |  |  |
|  | gb|DQ884132 | (3,5,7dpi)- | gb|ABA95869.1| Protein kinase domain, putative | Oryza sativa | 4.00E-06 | 72 |
|  | gb|DQ884131 | (BI,5,7dpi)- | ref|NP_915282.1| serine/threonine protein kinase | Oryza sativa | 2.00E-13 | 78 |
|  | gb|DQ884124 | (2,5,7dpi)+ | ref|NP_910025.1| receptor kinase | Oryza sativa | 2.00E-09 | 100 |
|  | gb|DQ884096 | (7dpi)+ | ref|XP_475069.1| methionine S-methyltransferase (EC 2.1.1.12) | Oryza sativa | 1.00E-06 | 100 |
|  | gb|DQ884095 | (7dpi)+ | dbj|BAD45924.1| auxin response factor | Oryza sativa | 2.00E-12 | 100 |
|  | gb|DQ884093 | (5,7dpi)- | gb|AAT77080.1| inositol phosphate kinase | Oryza sativa | 2.00E-13 | 90 |
|  | gb|DQ884078 | (5,7dpi)+ | dbj|BAD38550.1| patatin homolog | Oryza sativa | 3.00E-14 | 85 |
|  | gb|DQ884071 | (5,7dpi)- | gb|AAK52142.2| protein kinase | Oryza sativa | 6.00E-15 | 95 |
|  | gb|DQ884023 | (5,7dpi)- | dbj|BAD87380.1| calmodulin-binding family protein-like | Oryza sativa | 3.00E-17 | 98 |
| Transcription | |  |  |  |  |  |
|  | gb|DQ884130 | (BI,2dpi)- | gb|ABA92070.1| mRNA capping enzyme - like protein | Oryza sativa | 2.00E-15 | 100 |
|  | gb|DQ884128 | (7dpi)+ | gb|ABA91237.1| WRKY DNA binding domain, putative | Oryza sativa | 2.00E-17 | 100 |
|  | gb|DQ884120 | (5,7dpi)+ | ref|XP_479412.1| chromodomain-helicase-DNA-binding protein-like protein | Oryza sativa | 2.00E-24 | 100 |
|  | gb|DQ884119 | (2,5,7dpi)+ | gb|AAT28674.1| bZIP transcription factor | Oryza sativa | 4.00E-23 | 84 |
|  | gb|DQ884108 | (BI,2,5,7dpi)+ | ref|XP_477156.1| ABC1 family protein kinase-like protein | Oryza sativa | 5.00E-26 | 98 |
|  | gb|DQ884106 | (2,5,7dpi)+ | gb|AAL73487.1| repressor protein | Oryza sativa | 2.00E-28 | 100 |
|  | gb|DQ884077 | (5dpi)- | dbj|BAA08553.1| OSH45 | Oryza sativa | 2.00E-27 | 82 |
|  | gb|DQ884076 | (5,7dpi)+ | dbj|BAA08554.1| OSH45 | Oryza sativa | 8.00E-23 | 100 |
|  | gb|DQ884050 | (7dpi)+ | ref|XP_479418.1| zinc finger protein family-like | Oryza sativa | 5.00E-34 | 99 |
| Transport |  |  |  |  |  |  |
|  | gb|DQ884118 | (2dpi)+ | ref|NP_915325.1| ABC transporter | Oryza sativa | 2.00E-65 | 99 |
|  | gb|DQ884099 | (BI)+ | dbj|BAD45881.1| ionotropic glutamate receptor ortholog GLR6 | Oryza sativa | 4.00E-11 | 100 |
|  | gb|DQ884097 | (BI,2dpi)+ | ref|NP_914292.1| simiar to ATP-binding cassette, sub-family D, member 3 | Oryza sativa | 7.00E-06 | 100 |
|  | gb|DQ884094 | (7dpi)+ | gb|ABB47353.1| POT family, putative | Oryza sativa | 9.00E-05 | 95 |
|  | gb|DQ884087 | (2,5,7dpi)+ | gb|ABD36346.1| GTP-binding nuclear protein Ran | Bombyx mori | 4.00E-15 | 100 |
|  | gb|DQ884064 | (BI,2dpi)+ | gb|AAV59449.1| MRP-like ABC transporter | Oryza sativa | 2.00E-13 | 78 |
|  | gb|DQ884049 | (5,7dpi)+ | ref|XP_550309.1| band 3 anion transport protein -like | Oryza sativa | 1.00E-12 | 100 |
|  | gb|DQ884038 | (BI,2,5dpi)- | ref|NP_919551.1| LeOPT1 - oligopeptide transporter | Oryza sativa | 8.00E-42 | 88 |
|  | gb|DQ884036 | (BI,2,5dpi)- | gb|ABB47353.1| POT family, putative | Oryza sativa | 4.00E-05 | 100 |
| Retrotransposons | |  |  |  |  |  |
|  | gb|DQ884067 | (5,7dpi)- | ref|NP_918586.1| OsTATC | Oryza sativa | 2.00E-20 | 100 |
|  | gb|DQ884051 | (BI,2dpi)- | gb|ABA94469.1| hAT family dimerisation domain, putative | Oryza sativa | 5.00E-09 | 100 |
| Unclassified | |  |  |  |  |  |
|  | gb|DQ884123 | (2,5,7dpi)- | gb|ABA92061.1| hypothetical protein LOC_Os11g11000 | Oryza sativa | 1.00E-06 | 100 |
|  | gb|DQ884114 | (7dpi)+ | dbj|BAD89467.1| nuclear protein | Oryza sativa | 2.00E-17 | 100 |
|  | gb|DQ884109 | (5dpi)+ | dbj|BAD28362.1| hypothetical protein | Oryza sativa | 9.00E-69 | 91 |
|  | gb|DQ884105 | (2,5,7dpi)- | ref|XP_474301.1| OSJNBa0043A12.38 | Oryza sativa | 2.00E-28 | 89 |
|  | gb|DQ884101 | (5,7dpi)- | dbj|BAA02151.1| 21kd polypeptide | Oryza sativa | 1.00E-49 | 97 |
|  | gb|DQ884084 | (BI,2,5,7dpi)- | ref|XP_474856.1| OSJNBa0035O13.14 | Oryza sativa | 1.00E-42 | 100 |
|  | gb|DQ884081 | (5,7dpi)- | ref|XP_472763.1| OSJNBa0072F16.20 | Oryza sativa | 3.00E-07 | 100 |
|  | gb|DQ884080 | (5,7dpi)- | gb|ABA99609.1| hypothetical protein LOC_Os12g44170 | Oryza sativa | 5.00E-14 | 100 |
|  | gb|DQ884075 | (5,7dpi)- | ref|XP_473800.1| OSJNBb0015N08.12 | Oryza sativa | 2.00E-49 | 90 |
|  | gb|DQ884069 | (BI,2,5,7dpi)- | gb|ABA99439.1| hypothetical protein LOC_Os12g42990 | Oryza sativa | 1.00E-06 | 69 |
|  | gb|DQ884060 | (5,7dpi)- | ref|XP_473383.1| OSJNBb0038F03.7 | Oryza sativa | 6.00E-31 | 87 |
|  | gb|DQ884059 | BI- (2,5,7dpi)- | ref|XP_550567.1| SEU1 protein | Oryza sativa | 5.00E-10 | 68 |
|  | gb|DQ884045 | (5,7dpi)- | ref|XP_471056.1| OSJNBa0020P07.1 | Oryza sativa | 7.00E-51 | 100 |
|  | gb|DQ884043 | (5,7dpi)- | gb|ABA95762.1| expressed protein | Oryza sativa | 1.00E-09 | 97 |
|  | gb|DQ884030 | (5,7dpi)- | ref|XP_470024.1| unknown protein | Oryza sativa | 5.00E-11 | 100 |
|  | gb|DQ884028 | (7dpi)+ | ref|XP_472625.1| P0076O17.10 | Oryza sativa | 5.00E-09 | 100 |
|  | gb|DQ884074 | (7dpi)+ | gb|AAU44225.1| unknown protein | Oryza sativa | 6.00E-23 | 67 |
|  | gb|DQ884041 | (7dpi)+ | - | - | - | - |
|  | gb|DQ884138 | (5,7dpi)- | - | - | - | - |
|  | gb|DQ884137 | (5,7dpi)- | - | - | - | - |
|  | gb|DQ884134 | (7dpi)+ | - | - | - | - |
|  | gb|DQ884133 | (5,7dpi)+ | - | - | - | - |
|  | gb|DQ884129 | (5dpi)+ | - | - | - | - |
|  | gb|DQ884127 | (5,7dpi)- | - | - | - | - |
|  | gb|DQ884126 | (5,7dpi)+ | - | - | - | - |
|  | gb|DQ884121 | (BI,2,5dpi)- | - | - | - | - |
|  | gb|DQ884117 | (2,5,7dpi)+ | - | - | - | - |
|  | gb|DQ884116 | (BI)+ | - | - | - | - |
|  | gb|DQ884112 | (BI,2,5dpi)- | - | - | - | - |
|  | gb|DQ884110 | (7dpi)+ | - | - | - | - |
|  | gb|DQ884107 | (BI,2,5,7dpi)- | - | - | - | - |
|  | gb|DQ884102 | (5,7dpi)+ | - | - | - | - |
|  | gb|DQ884100 | (7dpi)+ | - | - | - | - |
|  | gb|DQ884092 | (BI,7dpi)+ | - | - | - | - |
|  | gb|DQ884091 | (BI,2,5dpi)- | - | - | - | - |
|  | gb|DQ884089 | (7dpi)+ | - | - | - | - |
|  | gb|DQ884088 | (7dpi)+ | - | - | - | - |
|  | gb|DQ884086 | (5,7dpi)+ | - | - | - | - |
|  | gb|DQ884085 | (5,7dpi)+ | - | - | - | - |
|  | gb|DQ884083 | (BI,2,5,7dpi)- | - | - | - | - |
|  | gb|DQ884082 | (5,7dpi)- | - | - | - | - |
|  | gb|DQ884072 | (7dpi)+ | - | - | - | - |
|  | gb|DQ884070 | (BI,2,5,7dpi)- | - | - | - | - |
|  | gb|DQ884068 | (5,7dpi)- | - | - | - | - |
|  | gb|DQ884065 | (5dpi)+ | - | - | - | - |
|  | gb|DQ884061 | (5,7dpi)- | - | - | - | - |
|  | gb|DQ884054 | (2dpi)+ | - | - | - | - |
|  | gb|DQ884053 | (5,7dpi)- | - | - | - | - |
|  | gb|DQ884052 | (7dpi)+ | - | - | - | - |
|  | gb|DQ884048 | (5,7dpi)+ | - | - | - | - |
|  | gb|DQ884046 | (5,7dpi)+ | - | - | - | - |
|  | gb|DQ884044 | (5,7dpi)+ | - | - | - | - |
|  | gb|DQ884042 | (7dpi)+ | - | - | - | - |
|  | gb|DQ884040 | (BI)+ | - | - | - | - |
|  | gb|DQ884039 | (BI,2,5,7dpi)- | - | - | - | - |
|  | gb|DQ884037 | (5,7dpi)- | - | - | - | - |
|  | gb|DQ884033 | (7dpi)+ | - | - | - | - |
|  | gb|DQ884032 | (5,7dpi)- | - | - | - | - |
|  | gb|DQ884031 | (5,7dpi)- | - | - | - | - |
|  | gb|DQ884029 | (5dpi)+ | - | - | - | - |
|  | gb|DQ884027 | (5dpi)+ | - | - | - | - |
|  | gb|DQ884026 | (7dpi)+ | - | - | - | - |
|  | gb|DQ884025 | (5,7dpi)- | - | - | - | - |

a: cDNA-AFLP fragment GenBank accession number; b: pattern of deregulation, + for up regulation, - for down regulation, the points of the kinetic showing a variation gene expression are indicated in brackets, BI for buffer inoculated control
